# Spontaneous liquid crystal and ferromagnetic ordering of colloidal magnetic nanoplates

**DOI:** 10.1038/ncomms10394

**Published:** 2016-01-28

**Authors:** M. Shuai, A. Klittnick, Y. Shen, G. P. Smith, M. R. Tuchband, C. Zhu, R. G. Petschek, A. Mertelj, D. Lisjak, M. Čopič, J. E. Maclennan, M. A. Glaser, N. A. Clark

**Affiliations:** 1 Department of Physics and Soft Materials Research Center, University of Colorado, Boulder, Colorado 80309, USA; 2 Advanced Light Source, Lawrence Berkeley National Laboratory, Berkeley, California 94720, USA; 3 Department of Physics, Case Western Reserve University, Cleveland, Ohio 44106, USA; 4 Jozef Stefan Institute, SI-1000 Ljubljana, Slovenia; 5 Faculty of Mathematics and Physics, University of Ljubljana, SI-1000 Ljubljana, Slovenia

## Abstract

Ferrofluids are familiar as colloidal suspensions of ferromagnetic nanoparticles in aqueous or organic solvents. The dispersed particles are randomly oriented but their moments become aligned if a magnetic field is applied, producing a variety of exotic and useful magnetomechanical effects. A longstanding interest and challenge has been to make such suspensions macroscopically ferromagnetic, that is having uniform magnetic alignment in the absence of a field. Here we report a fluid suspension of magnetic nanoplates that spontaneously aligns into an equilibrium nematic liquid crystal phase that is also macroscopically ferromagnetic. Its zero-field magnetization produces distinctive magnetic self-interaction effects, including liquid crystal textures of fluid block domains arranged in closed flux loops, and makes this phase highly sensitive, with it dramatically changing shape even in the Earth's magnetic field.

## Abstract (web summary)

Ferromagnetism has been known as a material property of solids since the time of the ancient Greeks. Here, Shuai *et al*. report that magnetic nanoplates suspended in a simple solvent can spontaneously align to form a ferromagnetic liquid, capable of both producing and sensing magnetic fields.

## Graphical abstract

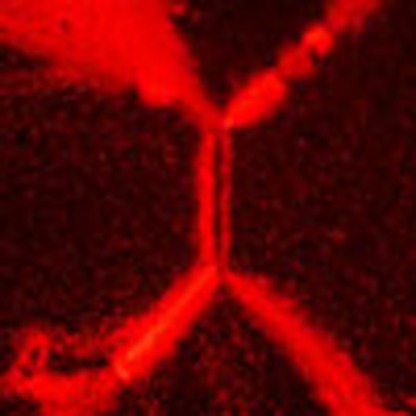

Colloidal suspensions of nanoparticles offer exciting and ever-expanding opportunities for creation and application of novel hybrid particle/fluid phenomena. A classic example is the paramagnetic ferrofluid, a colloidal suspension of ferromagnetic nanoparticles dispersed in isotropic solvent^1^,^2^. The ferrofluid particles disperse with random orientations but readily align and move in magnetic fields, resulting in dazzling hybrid behaviours, including radical changes in shape^3^,^4^ and magnetically controlled transport, which enable a variety of technical and biomedical applications^5^,^6^. A longstanding challenge has been to make fluids that are ferromagnetic, such that they exhibit spontaneous, equilibrium magnetic ordering in the absence of an applied field^7^,^8^,^9^,^10^,^11^. Such phases are conceivable because in such hybrid suspensions the dipole–dipole interaction energy between the supermolecular magnetic moments of neighbouring particles can be many times the thermal energy at room temperature^10^,^11^.

One approach to achieve ferromagnetism in colloidal fluids is to create stable, fluid suspensions of well-dispersed magnetic nanoparticles in isotropic solvent, and to design their mutual interactions to produce equilibrium, zero-field magnetization. Since ferromagnetic ordering implies orientational ordering of the particles, such fluid suspensions are also liquid crystals (LCs). Suspensions of magnetic nanoplates with moments normal to their planes are of particular interest to this study. One reason is that anisotropic steric interactions can lead to spontaneous LC ordering^12^, which have been demonstrated in various colloidal suspensions of nanoplates and nanosheets^13^,^14^. Meanwhile, the plate-like (as opposed to spherical or rod-like) shape promotes parallel rather than antiparallel orientation of the moments of neighbouring particles, making the interplate magnetic dipole interactions sufficient to stabilize ferromagnetic ordering, as recently demonstrated in dilute suspensions of nanoplates orientationally ordered by a thermotropic nematic LC host fluid^15^,^16^.

In our experiments, barium hexaferrite (BF) nanoplates are suspended in isotropic *n*-butanol and surfactant-stabilized to produce a system of functionalized nanoplates with weak electrostatic repulsion, strong and anisotropic steric repulsion and magnetic interaction. The electrostatic repulsion enables stable suspensions at essentially any concentration, including at high volume fractions where spontaneous LC ordering may occur. We observe nematic ordering of the nanosheets at volume fractions in the range where the Onsager model and subsequent theory and simulations predict an isotropic (Iso)–nematic transition for polydisperse, plate-like colloids^17^,^18^,^19^. This nematic is distinctly ferromagnetic, with a magnetization density two orders of magnitude larger than that reported in the previously found thermotropic nematic LC host (4-cyano-4′-pentylbiphenyl, 5CB) fluid system^15^.

## Results

### Isotropic to ferromagnetic nematic phase transition

**Figure 1:**
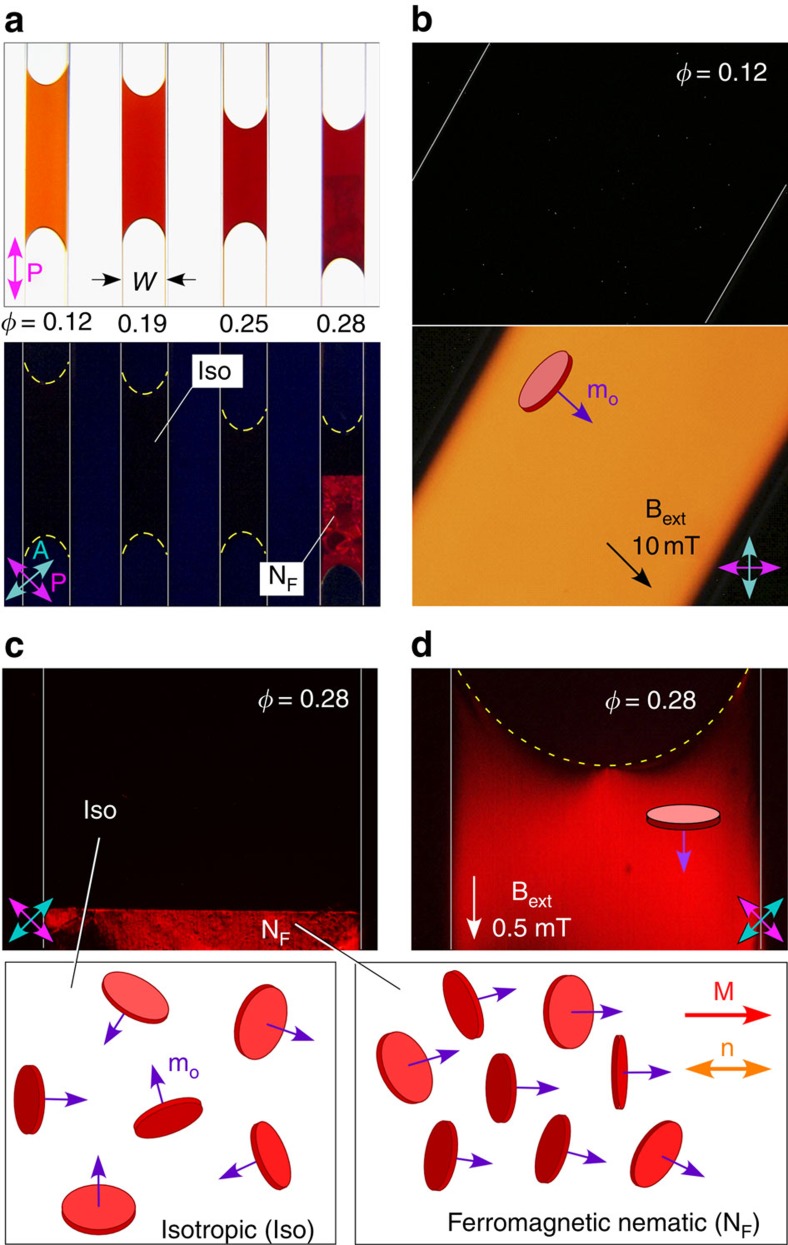
Liquid crystal ordering of barium ferrite nanoplatelets suspended in *n*-butanol. (**a**) Barium hexaferrite nanoplatelet suspensions viewed in transmitted light with optical polarization conditions indicated (Polarizer: magenta, **P**; analyser: cyan, **A**). Low-volume fraction suspensions (*φ*≲0.25) are isotropic (Iso), appearing dark between crossed polarizers. The orange/red colour is due to optical absorption by the nanoplatelets. At higher concentrations (*φ*≳0.28), a birefringent ferromagnetic nematic (N_F_) phase appears in the lower part of the cell. (**b**) An applied in-plane magnetic field **B**_ext_ induces birefringence in the isotropic phase, with the principal axes of the optical dielectric tensor along and normal to **B**_ext_ and the induced macroscopic magnetization density **M** parallel to **B**_ext_. (**c**) The N_F_ phase is separated gravitationally from the isotropic region by a sharp, horizontal interface. Equilibrium Iso and N_F_ structures deduced from birefringence and dichroism measurements are illustrated, where **m**_o_ is the nanoplate magnetic moment and **n** the director, indicating the local, mean nanoplate normal. (**d**) The Iso phase is magnetized and the Iso–N_F_ interface becomes continuous under 0.5-mT applied magnetic field. Samples are sealed in rectangular glass capillaries of thickness *L*=50 μm and width *W*=1 mm. The boundaries of the cells are indicated by the solid thin white lines. The air–liquid interfaces are indicated by the dashed yellow lines.

Suspensions of BF nanoplates (overall thickness *t*=7 nm, diameter *D*=48±21 nm, Supplementary Fig. 1) in *n*-butanol (BF/BuOH) are loaded in capillaries and observed at various magnifications using both unpolarized and depolarized transmitted light microscopy, which enabled visualization of their basic phase behaviour as a function of the volume fraction of the functionalized nanoplates, *φ*=*π*<*D*^2^*t>ν*/4, where *ν* is the number density (Fig. 1a). For *φ*<0.28, the suspensions are optically isotropic, giving excellent extinction for any sample orientation between crossed polarizer and analyser, and uniform transmission with only the polarizer, indicating that the particles are well dispersed and that the suspensions are optically homogeneous and isotropic on micron and larger scales. The characteristic orange–red colour is due to nanoplate absorption (Supplementary Fig. 2). The isotropic suspensions are stable, with only weak gravitational concentration gradients developing over periods of hundreds of days (Supplementary Fig. 3), consistent with an estimated Perrin length *l*_P_=*k*_B_*T*/Δ*ρ*_LS(_*π*<*D*^2^*t*>/4)*g*∼1 cm (Δ*ρ*_LS_=mass density difference between nanoplates and solvent, *g*=9.8 m s^−2^).

With the application of an external magnetic field **B**_ext_, the Iso suspensions exhibit induced birefringence, with significant optical transmission between crossed polarizers when *B*_ext_∼10 mT is applied in the plane of the cell at an angle of 45° to the polarizer, **P** (Fig. 1b). This induced birefringence is spatially homogeneous, further evidence that the nanoplate dispersion is uniform on the optical scale. The direction with lowest refractive index is found by using a Berek optical compensator to be parallel to **B**_ext_. The Iso suspensions also exhibit magnetically induced optical dichroism, with the absorbance largest for optical polarization parallel to the planes of the nanoplates (Supplementary Fig. 2). Thus, the magnetized isotropic phase has field-induced, uniaxial nematic ordering of the plates, with the nematic director **n**(**r**), the unit vector along the local, the mean orientation direction of the nanoplate normals, aligned parallel to **B**_ext_, along the induced magnetization density **M**. The induced nematic has negative birefringence Δ*n*(*B*_ext_)=*n*_||_−*n*_⊥_<0 and a visible light-dichroic ratio *ζ*(*B*_ext_)=OD_||_/OD_⊥_<1 (where || (⊥) indicates orientation parallel (normal) to **n**).

At higher concentrations (*φ*≳0.28), fresh suspensions begin to sediment higher-density domains at the bottom of the capillary that are birefringent even in the absence of applied magnetic field. Over periods of hours to days, a well-defined, horizontal, planar interface between the birefringent region and the upper isotropic phase develops. The birefringent region, if left unperturbed, gradually develops LC-like textures with uniform domains that appear to grow in size and become more ordered with the passage of time (Fig. 1a,c, below the interface, and Supplementary Fig. 4). We identify this behaviour as a two-phase coexistence, with the denser phase sedimented from the less-dense Iso phase by gravity.

Further insight into the ordered phase can be obtained by observing the Iso–N_F_ interface with an external field applied, where we see that an initially sharp interface formed in the absence of field (Fig. 1c) begins to deform and move into the Iso region as **B**_ext_ is increased. For sufficiently large field (*B*_ext_∼0.5 mT), the ordered phase reorients to have the director along **B**_ext_, nematic order induced in the Iso phase becomes comparable to that of the ordered phase and the interface becomes continuous (Fig. 1d), qualitatively as described by the mean-field model of a field-induced isotropic/nematic transition of Fan and Stephen^20^. Induction of nematic order requires larger **B**_ext_ as the interface is pushed further up in the cell because the number density of nanoplates in the Iso region decreases with increasing height (Supplementary Fig. 3). If the applied field is removed, the interface reforms and returns to the state of Fig. 1c. These responses to changing field take several minutes to hours to complete because the distribution in the number density of plates must also change to re-establish a uniform chemical potential of the plates in the colloidal system. The field-induced continuity provides evidence that the birefringent phase below the interface is identical to that induced in the Iso phase, a uniaxial, magnetically polar nematic, having the first- and second-rank nanoplate orientational order parameters *Q*_1_ and *Q*_2_ simultaneously non-zero (ferromagnetic nematic, N_F_). If **B**_ext_ is instead reduced to a small but non-zero value, the Iso–N_F_ interface was also found, unexpectedly, to exhibit a field-induced spiking instability, discussed in detail below.

To assess the nature of the magnetic ordering in the vicinity of the Iso–N_F_ transition, we measured and analysed Δ*n* induced by an external magnetic field *B*_ext_, in effect probing the field-induced, uniaxial orientational order in the Iso and N_F_ phases (Supplementary Fig. 5). Analysis including the effects of polydispersity of the response of extremely low-concentration suspensions (*φ*=0.005) gives an estimate of the mean nanoplate magnetization of *m*_o_=2 × 10^−18 ^A m^2^ (Supplementary Note 1), which is very close to the value obtained from aligned and dried BF nanoplates with a mean diameter of 70 nm (ref. 15). As *φ* is increased, the initial (low-field) magnetic susceptibility, *χ*(*φ*), of the Iso phase, derived from the measurements of *Q*_2_=Δ*n*/Δ*n*_sat_, where Δ*n*_sat_ is the saturated birefringence, shows a substantial growth. Comparison shows that the standard Langevin–Weiss mean-field model^21^ significantly overestimates the growth of *χ*(*φ*) and predicts the Iso–N_F_ transition near *φ*=0.09, a much lower value than observed. However, treating the orientationally diffusing nanoplates of the Iso phase effectively as a system of dipolar hard spheres and applying models that include interparticle correlations^11^ gives a reasonable qualitative description of the Iso phase susceptibility (Supplementary Note 2).

**Figure 2:**
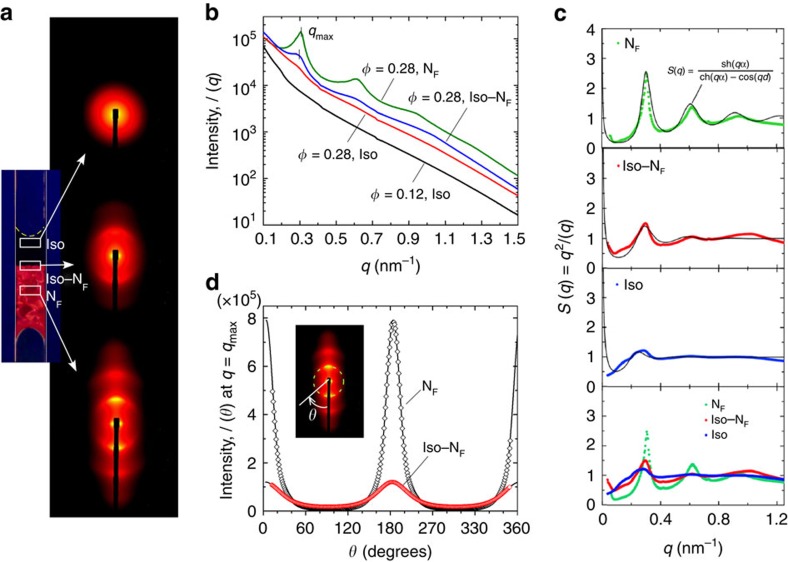
Synchrotron X-ray diffraction of BF/BuOH magnetic nanoplatelet suspensions. A suspension with *φ*=0.28 was filled into a flat capillary of thickness *L*=50 μm and width *W*=1 mm and sedimented for 1 day, after which it developed an Iso–N_F_ interface. (**a**) 2D diffraction patterns measured at the three heights in the sample indicated at left by the white boxes representing the beam size and location. *Location Iso*: immediately below the air–liquid interface; *Location Iso–N*_*F*_: immediately above the Iso–N_F_ interface; *Location N*_F_: in the N_F_ phase. (**b**) Diffraction intensity versus *q* obtained by circular integration of the 2D images in **a**. The integrated diffraction from a *φ*=0.12 sample is shown for comparison. This suspension is uniformly isotropic and the scattering is featureless. (**c**) Structure factor *S*(*q*)=*q*^2^*I*(*q*) at the three locations probed in the *φ*=0.28 cell. The position of the first diffuse peak moves from *q*_max_=0.30 nm^−1^ in the Iso–N_F_ region to 0.25 nm^−1^ in the Iso region, corresponding to an ∼15% density difference between the two locations, consistent with Supplementary Fig. 3. The solid curves are a fit to the equation shown, modelling the layer correlations in a suspension of thin plates, where *α* is the average of the magnitude of the orientation difference between neighbouring nanoplates. (**d**) Diffraction intensity *I*(*θ*) along the circle passing through the first diffuse peak for the Iso–N_F_ and N_F_ locations, respectively. This angular distribution is fitted by the function *I*(*θ*)=∑_*n*_ *p*_2*n*_cos^2*n*^*θ* (solid lines), where *θ* is the azimuthal angle as illustrated in the inset.

The LC order of the N_F_ phase was characterized by synchrotron X-ray diffraction. A high-concentration sample (*φ*=0.28) was kept vertical for several days until it developed a clear Iso–N_F_ interface. As shown in Fig. 2a, with the beam intersecting the upper part of the Iso part of the sample (*Location Iso*), the diffraction pattern is circularly symmetric, demonstrating the isotropic nature of the sample. When the beam is incident on the two-phase interface (*Location Iso–N*_*F*_), a pair of diffuse arcs appears, with a noticeable isotropic background. The higher-density phase region (*Location N*_*F*_) gives multiple diffraction arcs. If the diffracted signal is integrated along concentric circles around the beam, we obtain the dependence of scattering intensity versus wavevector *q* shown in Fig. 2b. The diffraction arcs are broad, indicating short-ranged lamellar correlations characteristic of a nematic phase^22^, with the peaks at almost the same locations in both regions of the sample. Fitting the structure factor *S*(*q*)=*q*^2^*I*(*q*) with the function *S*(*q*)=sh(*qα*)/[ch(*qα*)−cos(*qd*)] (*α* is the average of the magnitude of the orientation difference between neighbouring plates), describing lamellar correlations in an array of plates, gives peaks in the pair correlation function at *d*=20.0, 20.9 and 22.1 nm for the N_F_, Iso–N_F_ and Iso regions, respectively (Fig. 2c), showing that the difference in the number density between the two phases is small. The azimuthal distributions of the first-order diffraction intensities at *Location Iso–N*_*F*_ and *Location N*_*F*_ are plotted in Fig. 2d. Analysis of the azimuthal distributions of the diffraction intensity gives a second-rank order parameter *Q*_2_=0.8 (Supplementary Note 3).

### Equilibrium magnetic domain structure in a thin capillary

**Figure 3:**
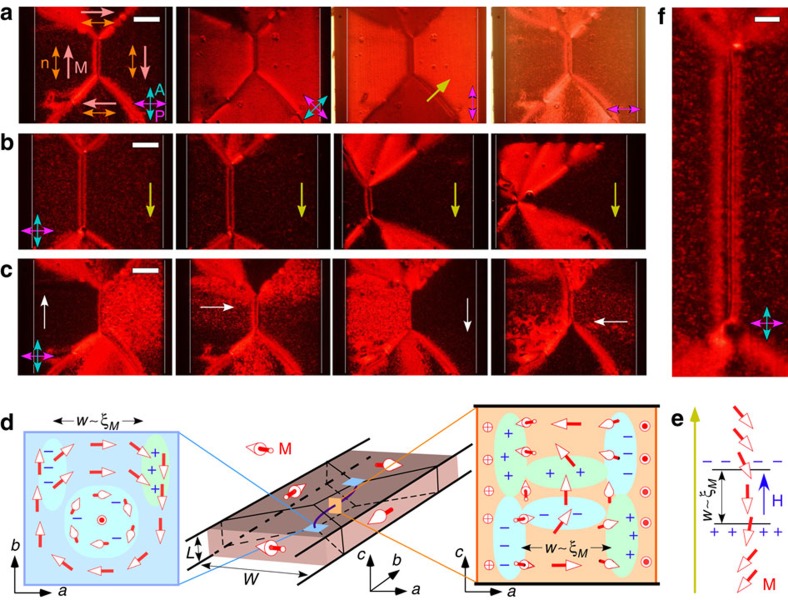
Equilibrium ferromagnetic nematic flux loop texture in a thin capillary. (**a**) Orientational domain textures in the N_F_ phase in a cell equilibrated in the absence of applied magnetic field viewed in transmission with optical polarization conditions [**P**, **A**] indicated. Uniform domains, with magnetization **M**(**r**) orient in a discontinuous loop to minimize magnetic charge at the domain boundaries. The domains extinguish wherever the director field **n**(**r**) is parallel or normal to crossed polarizers. Rotating the polarizers confirms that the domains are birefringent. Optical dichroism is revealed with **P** only, the nanoplate absorbance being larger for **P** parallel to the nanoplate planes (normal to **n**). (**b**) Applying *B*_equil_ (yellow arrows; from left: *B*_equil_=0.03 mT, 0.04 mT, 0.08 mT and 0.10 mT) induces domain wall displacement, allowing the domain with **M** parallel to **B**_equil_ to grow in area. (**c**) A small transient field (white arrows; *B*_trans_∼0.02 mT) applied parallel to **M** makes the orientation within that domain more uniform (darker). **B**_trans_ applied antiparallel to **M** produces a random texture of brighter, reorienting subdomains. (**a**–**c**) Scale bar, 200 μm. The cells are 1 mm in width, with the boundaries indicated by thin white lines. (**d**) An illustration of the twist wall passing along the cell centreline from one cell surface to the other. (**e**) Structure of the splay/bend/splay domain boundaries interior to the cell, along the line indicated by the olive arrow in **a**, stabilized by a balance of LC bend elasticity and magnetic charge attraction. (**f**) Magnified image of the +1 escaped twist wall in **n**(**r**) and **M**(**r**) imposed by the loop structure of **M**(**r**). Scale bar, 50 μm. *L*=50 μm, *W*=1 mm.

The response of the higher-density phase to an applied magnetic field was studied in some detail. This phase is a low-viscosity fluid that flows readily if the capillary is tilted and anneals if left undisturbed into domains that are several millimetres across and span the 50-μm thickness of the capillary (Fig. 3 and Supplementary Fig. 4). Cells were equilibrated over periods of up to several days with external static fields **B**_equil_. If *B*_equil_=0, obtained by locally cancelling the Earth's magnetic field and other stray fields, a characteristic texture of large, distinctly dichroic and birefringent domains, which divide the cell into almost uniform regions of different orientations separated by sharp domain boundaries as shown in Fig. 3, consistently develops. Measurement of the dichroism and birefringence of these domains and applying the relationship between **n**, Δ*n* and *ζ* discussed above in connection with the Iso phase show that the nanoplate director **n**(**r**) is oriented as indicated in Fig. 3a. If **B**_equil_ is applied parallel to **n** in the left- and right-hand domains in Fig. 3a, then one of these domains (in this case the left domain) shrinks and the other expands (Fig. 3b). If the sign of **B**_equil_ is reversed, then the domain growth/shrinkage is also reversed. The field-induced displacement of the inversion walls is plotted in Supplementary Fig. 6. These walls behave as a system of lines with inherent tension that adopt a geometry minimizing their net length while mediating the changes in director orientation. The field-induced wall displacements are a consequence of stresses that tend to change the domain areas according to the sign of the magnetic energy density *U*_M_=−**M·B**_equil_. The linear response of the displacement at low fields is a definitive evidence for a magnetization, **M**, that is finite and independent of applied field strength around *B*_equil_=0. The field-induced motion of the domains walls is maximal for **B**_equil_ in the **n** direction, showing that **M** is along **n** and uniquely determining its sign, as indicated in Fig. 3a. Equilibration in a finite field, such as the Earth's magnetic field, *B*_equil_∼0.05 mT, produces arrays of similarly structured domains that are more complex (Supplementary Fig. 4).

The domain magnetization is also evident from the internal response of the domains to applied fields **B**_trans_ changing over periods of several seconds. The big domains in the cell are observed to have a static, grainy texture of subdomains ∼10 μm in size in which the in-plane orientation varies around the mean by a few degrees. Applying **B**_trans_ parallel to **M** within a domain produces a continuous enhancement of the extinction of that domain when viewed between crossed polarizers as the magnitude of the applied field is increased (Fig. 3c). The field applies torques to the nanoplates that tend to reduce the orientational fluctuations of the director field **n**(**r**), orienting the plates more parallel on average to **B**_trans_ and thereby increasing *Q*_2_. Applying **B**_trans_ antiparallel to **M**, on the other hand, generates torques that tend to enhance the orientational disorder of the nanoplates, producing, above a certain threshold *B*_trans_∼0.005 mT, an instability marked by the appearance of a speckle-like pattern of birefringence generated by an array of subdomains of **M**(**r**) that have rotated away from the field direction in random directions and through a distribution of angles. Since these subdomains are substantially smaller than the cell thickness, they must be a response to static fluctuations and thus to quenched disorder in the local **M**,**n** fields. Strong magnetic self-interactions lead to the local expulsion of magnetic charge and the creation of subdomains responding as independent units to *B*_ext_. Since these changes take place at values of *B*_ext_ much smaller than the internal field (*B*_M_∼40 mT, see Supplementary Note 4), the response appears to be a soft deformation mode in the frozen transverse fluctuations of **M**. This behaviour is quite different from the Fréedericksz transitions found in the BF/5CB system^23^.

This dependence of the domain response on the sign of **B**_trans_, combined with imaging of the dichroism and birefringence-based optical textures, enables detailed mapping of **M**(**r**) and **n**(**r**) in any domain pattern. **M**(**r**) is found to be always parallel to **n**(**r**), which is consistent with the nanoplate magnetic moments being along their normals, and with the observed domain wall movements in applied fields. Importantly, the domain magnetization is non-zero in the absence of any applied magnetic field, as an equilibrium property of the phase.

The textural features and response to an applied field of the BF/BuOH ferromagnetic nematic phase may be understood as phenomena resulting from the combined effects of magnetostatic and nematic elastic energies. The interplay of Frank elasticity and magnetostatic self-interactions is expressed through the characteristic length 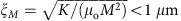 (*K* is the LC Frank elastic constant in the one-constant approximation, see Supplementary Fig. 7 and Supplementary Note 4), which for the BF/BuOH N_F_ phase is small compared with any cell dimension. Generally, the imperative produced by this condition is to reduce magnetostatic energy by reducing the excess internal and external magnetic fields generated. A distortion such as splay of **M**(**r**) that produces bulk magnetic charge of a particular sign [*ρ*_m_(**r**)=−∇**·M**(**r**)] must necessarily also produce the same quantity of opposite charge elsewhere in the bulk or at the interfaces with non-magnetized material [*ρ*_s_(**r**)=**M**(**r**)**·*****s***(**r**)]. Therefore, the drive for energy reduction leads either to non-splayed regions of **M**(**r**) tangent to boundaries, which produce minimal magnetic charge, or to orientational variation of **M**(**r**) such that the positive and negative magnetic charges generated are located as close as possible to each other, a separation that is at least *ξ*_M_ because of the LC elasticity (Supplementary Fig. 7). The resulting structure of uniform domains separated by distinct walls is a general feature of the BF/BuOH textures reported here. However, in systems where *ξ*_M_ is much larger than the cell dimensions, such as in the case of BF/5CB, the magnetic self-interactions affect the texture only weakly (Supplementary Note 5).

Our observations indicate that the N_F_ region in the flat capillary cells is magnetized with **M** preferentially parallel to the largest cell face (Fig. 3d). This is expected, considering that, for a slab of infinite area, the magnetic energy of a uniform **M** field-free to orient in any direction is lowest when **M** is in the plane of the slab (Supplementary Note 4). The response of the capillary cells to an applied field confirms this confinement effect (Supplementary Fig. 8), showing that, while fields of less than 0.2 mT were needed to induce a complete in-plane alignment of the entire sample, in the case of perpendicular field there is little observable response until *B*_ext⊥_ reaches ∼10 mT, and extinguishing regions first appear at ∼20 mT, indicating a domain-mediated transition orienting **n** normal to the cell face.

The equilibrium in-plane texture in the absence of external applied fields can be understood by approximating the N_F_ region as a two-dimensional (2D) slab the thickness of the cell, in which **n**(**r**) is a 2D field, uniform along the cell normal, **c** (Fig. 3d), and assuming that boundary conditions force **n**(**r**) to be parallel to the edges of the cell, which eliminates magnetic charge on the surfaces. **M**(**r**), thus constrained to be parallel to the **a**,**b** plane in Fig. 3d, adopts an equilibrium in-plane texture that is determined only by the in-plane sample shape and the magnetoelastic behaviour. In the case of rectangular cells with planar boundaries, the equilibrium configuration is a loop of uniform domains of **M** separated by sharp splay/bend/splay walls, such as that shown in Fig. 3e and Supplementary Fig. 7, forced into the texture of Fig. 3a by the bends of **M**(**r**) at the corners of the rectangle. These walls are observed to have an optical-resolution (micron scale or smaller) linear core and a diffuse width comparable to the cell thickness. Figure 3e shows that the deformation of **M**(**r**) through a splay/bend/splay wall generates lines of opposite magnetic charge, the attraction of which favours narrow walls. The Frank elastic energy associated with bend of the director **n**(**r**), on the other hand, favours broad walls, to reduce the curvature of the director reorientation at the wall centre^24^. Using *K*_S_∼6*k*_B_*T/D*=5 × 10^−13^ N from a numerical simulation^25^, this balance yields an equilibrium wall core structure of width *w*∼*ξ*_M_<1 μm, which is smaller than the optical resolution. This scenario is an example of ‘orientational fracture', similar to that found in −1 topological defects in the director field structure and textures of thermotropic ferroelectric smectic C LCs with large permanent polarization^26^, as detailed in Supplementary Note 6.

The loop geometry leads to an overall topological disclination line defect of strength +1 in the **M**,**n** fields of the sample. In the centre of the cell we observe a linear boundary between domains of antiparallel **M** (Fig. 3a–c), which higher magnification imaging shows to be a +1 disclination line singularity in the **M**,**n** fields (Fig. 3f) that starts on one cell surface at a point where three domains meet (blue square) and ends on the other cell surface at the other such point, as shown in Fig. 3d. This defect can be constructed by starting with a +1 bend disclination line (blue box) running along **c** from one cell face to the other (required by the boundary conditions on the cell edges) and then sliding the intersection points on the cell surfaces to the locations where the three domains meet (blue squares). This defect will ‘escape' into the third dimension, forming a continuous twist wall in the cell centre (orange square) confined by the mutual attraction of the opposite-signed magnetic space charge.

The equilibrium, field-free domain pattern of **M**(**r**) in Fig. 3 resembles that of the closed flux loops found in solid ferromagnetic single crystals^27^, known to be an organization of their magnetization field **M**(**r**) that reduces magnetic charge *ρ*_m_(**r**)=−∇**·M**(**r**) and thereby the magnetic field and energy external to the sample. In crystals, such patterns are achieved with the aid of the crystal anisotropy, which pins **M**(**r**) to certain ‘easy' symmetry directions. In ferromagnetic LCs, however, there is no underlying lattice to stabilize discontinuous reorientation of **M**(**r**). Equilibrium domain patterns are established by the sample shape and by magnetoelastic interactions described in Supplementary Note 4. An interesting special case where the magnetization can vary continuously with no need for domain walls is the ring cell—for example, one shaped like a flat washer or a racetrack. The equilibrium **M**(**r**) here is everywhere parallel to the cell boundary, a geometry with continuous bend depending only on radius.

### Spikes at the Iso–N_F_ interface

**Figure 4:**
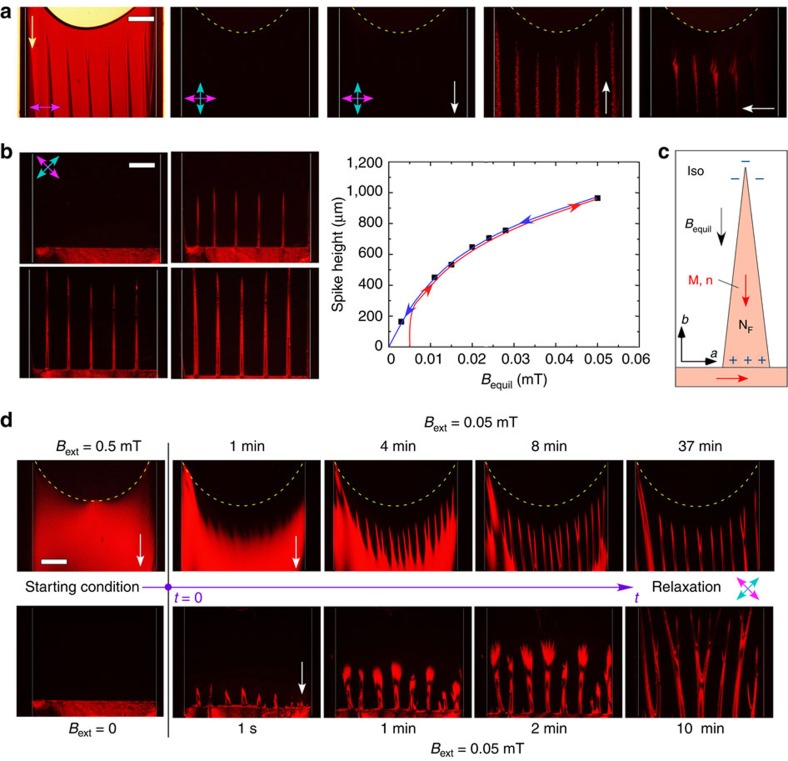
Ferromagnetic nematic spikes at the BF/BuOH isotropic/nematic interface. (**a**) Spikes obtained by equilibrating a *φ*=0.28 suspension in a rectangular capillary in an external field (yellow arrow, *B*_equil_=0.05 mT) for 2 days. The optical polarization conditions [**P**, **A**] are indicated. The reaction of the spikes to transient fields (white arrows, **B**_trans_∼0.02 mT) indicates that **n** and **M** in the spikes are in the same direction as **B**_equil_. (**b**) Spike height versus *B*_equil_. The spike growth is limited by their penetration into the lower-susceptibility Iso phase in the top of the cell. (**c**) Sketch of ferromagnetic spike at the Iso–N_F_ phase boundary showing magnetization and magnetic charges. (**d**) Spike dynamics. Application of a large external field (*B*_ext_=0.5 mT) induces saturated **M**, except near the Iso–air interface. On reducing *B*_ext_ to 0.05 mT, the N_F_–Iso interface reforms initially with an undulation instability, which then evolves into spikes. After application of an external field *B*_ext_=0.05 mT to an initially smooth interface formed at *B*_ext_=0, on interfaces instability appears and grows into spikes. The cells are 1 mm in width with boundaries indicated by the thin white lines. Scale bar, 200 μm.

A dramatic manifestation of the fluid ferromagnetic ordering in the BF/BuOH system is the interfacial spiking behaviour shown in Fig. 4 and Supplementary Fig. 4. In fresh cells, the Iso–N_F_ interface is initially flattened by gravitational sedimentation of domains of N_F_ (Fig. 1c). However, applying an external magnetic field of *B*_equil_≳0.005 mT normal to this interface causes a quasi-periodic array of birefringent spikes to grow, which equilibrate to a field-dependent height above the interface over the course of a day (Fig. 4a,b). Exposure of the spikes to transient fields of few-second duration shows that they are nematic and ferromagnetic, with magnetization parallel to the spike axis along **B**_equil_, with a field response very similar to that of the bulk N_F_ domain.

The spike geometry appears to be a soliton-like response of the interface that enables the magnetization of a ferromagnetic fluid to align with **B**_ext_. The extended shape of the spikes, with **M**(**r**) oriented along the spike axis, minimizes the generation of magnetic charge, with the remnant opposite magnetic charges on opposite ends of the spikes, indicated in Fig. 4c, decreasing as the spikes become longer and narrower. Each spike is effectively a small bar magnet, making the spikes mutually repulsive, as is the case for similarly aligned bar magnets placed next to one another. The spikes are roughly conical in shape, becoming narrower in both the **a** and **c** directions with increasing distance from the interface, the latter case evident from the birefringent interference extinctions at places where the phase shift is an odd multiple of π.

Spikes tend to grow in an external field if the magnetic energy gain is larger than the cost of increasing the interface area, for example, for a cylindrical spike of radius *R*∼25 μm when *B*_ext_>2*σ*_IN_/*RM*∼0.02 mT. As discussed in connection with the Iso phase, application of a sufficiently large *B*_ext_ normal to the interface of an equilibrated sample induces nematic order everywhere, expanding the strong ordering of the bulk nematic well into the Iso region for *B*_ext_≳0.5 mT (Fig. 1d). On the removal of *B*_ext_ from such a sample previously equilibrated with spikes, both the interface and the spikes evolve quickly over a period of a few minutes, as shown in Fig. 4d, to an instability of interface position. This experiment shows that the spike length at a given field is limited by the decrease in the susceptibility to magnetic ordering with distance from the Iso–N_F_ interface.

We propose that spike formation can be understood qualitatively as a novel type of nonlinear capillary instability of the sort produced in fluid interfaces when they become electrically or magnetically charged by the application of an external field^3^,^5^,^28^. As discussed above, in a ferromagnetic fluid, the permanent magnetization **M** is parallel to the interface when *B*_ext_=0, but, as **M** rotates in response to an applied field normal to the interface, it develops a normal component **M**_⊥_=**B**_ext_/*μ*_o_. This, in turn, produces magnetic charge *B*_ext_/*μ*_o_, and an effective magnetization energy density for driving the instability. Near the threshold, the instabilities are periodic with wavelength *λ*_G_=2*π*·*l*_G_, where 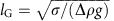 is the gravitational capillary length (*σ*=surface tension, Δ*ρ*=mass density difference between Iso and N_F_ phases). The associated increase in capillary energy density^3^ is 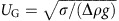. Taking *σ*_IN_=0.1*k*_B_*T*/*D*^2^∼10^−7 ^J m^−2^ as an estimate of the Iso–N_F_ interfacial tension^29^,^30^, we find *λ*_G_∼200 μm, comparable to the observed spike spacing, and *U*_G_∼0.002 J m^−3^, energy that is provided by the applied field.

## Discussion

A variety of observations discussed above indicate that the Iso and N_F_ phases are equilibrium dispersions of individual nanoplates. First, the magnitude of the magnetization of the orientable unit producing the magnetic birefringence in the Iso phase is near that of a single plate, which must therefore act as thermalized individuals. Second, the Iso–N_F_ phase coexistence appears at *φ*∼0.28, equivalent to a scaled density of *ν*<*D*^3^>=3.3, a volume fraction value where nematic ordering due to the steric and charge interactions of single nanoplates is expected^18^,^19^,^31^. Third, X-ray diffraction from oriented monodomains of the NF phase reveals several diffuse quasi-Bragg reflections from short-ranged lamellar ordering with a layer spacing of *d*=20 nm (Fig. 2). Assuming that the nanoplates form nearly continuous sheets, that is, the volume fraction *φ*=0.28 is determined by the spacing between the neighbouring plates, it gives a plate thickness *t*=*φd*=5.6 nm. This is comparable to the 7-nm thickness of a single nanoplate, providing further evidence that the BF/BuOH system is a suspension of single plates dispersed in equilibrium and distinguishing the ferromagnetism observed here from that of the non-ergodic aggregation of magnetic particles^8^,^9^. Finally, observations above suggest that magnetic dipolar and steric quadrupolar interactions, tending to stabilize *Q*_1_ and *Q*_2_, respectively, contribute to the ordering transition.

Several theoretical treatments of ordering transitions in systems with both dipolar and quadrupolar interactions have explored the interplay of the ordering of *Q*_1_ and *Q*_2_, in particular the Sivardiere and Blume (SB) Ising model in the molecular field approximation^32^, and the Baus and Colot (BC) density functional theory of a system of dipolar hard ellipsoids^33^. Despite the very different nature of these two systems, the SB and BC phase diagrams, shown in Supplementary Fig. 9, exhibit some key qualitative common features relevant to the BF/BuOH system. Both the SB and BC show that the two types of ordering are surprisingly weakly coupled, with the isotropic/nematic quadrupolar transition independent of the dipole interaction until the latter becomes strong enough for dipole ordering to occur at a smaller density than the quadrupolar ordering. On the other hand, the dependence of the (isotropic or nematic)/N_F_ transition on the dipole strength is only weakly affected by whether the quadrupolar ordering has taken place or not. Our independent assessment above of the proximity of the two transitions at *φ*∼0.28 leads us to conclude that on these phase diagrams the BF/BuOH system is in the vicinity of the tricritical point where, with an increasing dipole strength, the nonferromagnetic nematic phase has just disappeared. This is a place where the remnant Iso–N_F_ transition will be first order, and where there will be significant short-ranged quadrupolar ordering (nonpolar alignment of the nanoplates) and thus an enhanced susceptibility for inducing *Q*_2_.

Those familiar with smectic LCs may recognize the similarity of the phase diagram in Supplementary Fig. 9 to McMillan mean-field isotropic/nematic/smectic A model, with ferromagnetic ordering replacing the appearance of smectic A layering^34^. McMillan's distinct mean-field approach is thus yet another route to the generic dipolar/quadrupolar phase diagram. The Landau model of Palffy-Muhoray, Lee and Petschek also produces a similar Iso, nematic, N_F_ phase diagram^35^.

## Methods

### Preparation of the BF suspensions

BF nanoplatelets were suspended in *n*-butanol to form a stable colloidal suspension following a previously established method^36^,^37^. Briefly, BF nanoplatelets with the nominal composition BaFe_11.5_Sc_0.5_O_19_ were synthesized hydrothermally at 240 °C, with dodecylbenzenesulphonic acid added as a surfactant. The nanoplatelets were subsequently washed with water and nitric acid. To increase the adsorption of the dodecylbenzenesulphonic acid on the nanoplatelets, they were suspended in an HNO_3_ solution (pH=1.5) and heated at 100 °C for 2.5 h. After this, the nanoplatelets were washed again with water and acetone, dried in air at 60 °C and dispersed in *n*-butanol using probe sonication. The final volume fraction of platelets *φ*=0.003 was obtained by concentrating the initial suspension (*φ*=0.001) in a rotary evaporator. The functionalized nanoplatelets have an average magnetic core thickness of 5 nm, with 1 nm of surfactant on each side, and a mean diameter of *D*=48 nm with a s.d. of 21 nm, which gives an aspect ratio of ∼7. Suspensions prepared at the volume fraction obtained in the synthesis of the platelets, *φ*=0.003, formed an isotropic liquid. Higher concentrations were prepared by compression, using ultracentrifugation at 160,000*g* for 40 min. The clear supernatant was then removed and the remaining concentrated suspension, at *φ*∼0.1, homogenized. Repeated addition of low-concentration suspension followed by recompression eventually yields suspensions with desired higher concentrations. The suspensions, loaded into the 50 μm thick × 1 mm wide × 25 mm long sample cavity of rectangular capillaries, flame-sealed and held vertically, are stable for months under ambient conditions, showing no sedimentation or aggregates visible in the optical microscope.

### Synchrotron X-ray experiment

The orientational order of the nanoplates in a phase-separated suspension was studied using synchrotron X-ray diffraction performed on Beamline 7.3.3 of the Advanced Light Source, Berkeley, CA, USA. The suspensions were contained in rectangular (0.05 × 1.0 mm^2^) borosilicate glass capillaries. The sample was probed at multiple locations in the capillary with a 10-keV synchrotron X-ray beam with a Mo/B_4_C double-multilayer monochromator and a beam at the sample ∼700 μm wide by 300 μm high.

## Additional information

**How to cite this article:** Shuai, M. *et al*. Spontaneous liquid crystal and ferromagnetic ordering of colloidal magnetic nanoplates. *Nat. Commun.* 7:10394 doi: 10.1038/ncomms10394 (2016).

## Supplementary Material

Supplementary Information Supplementary Figures 1-9, Supplementary Notes 1-6 and Supplementary References
