# Supplementary material for: Spontaneous liquid crystal and ferromagnetic ordering of colloidal magnetic nanoplates
Source: Nat Commun. 2016 Jan 28;7:10394. doi: 10.1038/ncomms10394 (PMC4738347; doi:10.1038/ncomms10394)
Supplement: Supplementary Information — Supplementary Figures 1-9, Supplementary Notes 1-6 and Supplementary References [file ncomms10394-s1.pdf]

SUPPLEMENTARY FIGURES

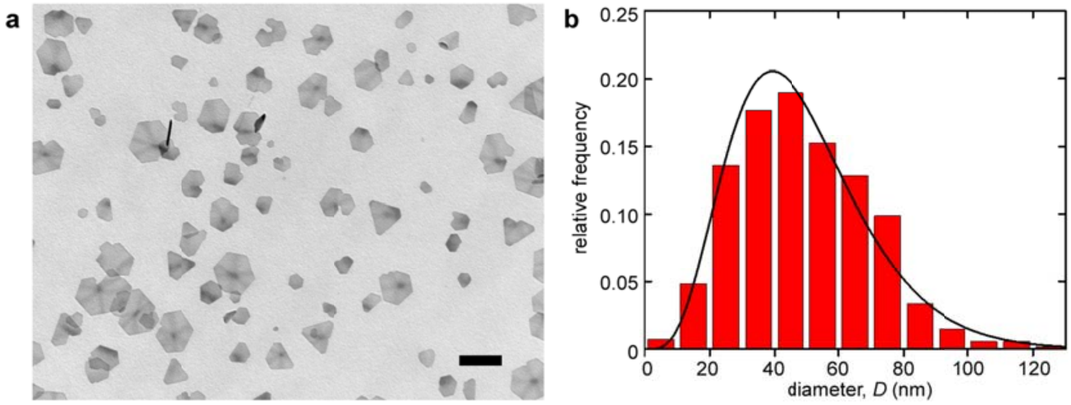

Supplementary Figure 1: Disk-shaped BF nanoplates and their diameter distribution.

(**a**) Transmission electron microscope (TEM) image of functionalized BF nanoplates. Scale bar: 100 nm. (**b**) Diameter distribution of the BF nanoploates based on statistical analysis of 500+ nanoplates from TEM images. The measured distribution is fitted by the gamma distribution function, $f(D;\kappa,\tau) = D^{\kappa-1}e^{-D/\tau}\big/[\tau^{\kappa}\Gamma(\kappa)]$ (solid line), where $\Gamma(\kappa)$ is the gamma function evaluated at κ, and κ and τ are fitting parameters (κ = 5.2 and τ = 9.2). The mean diameter of the nanoplates is 48 nm with a standard deviation of 21 nm.

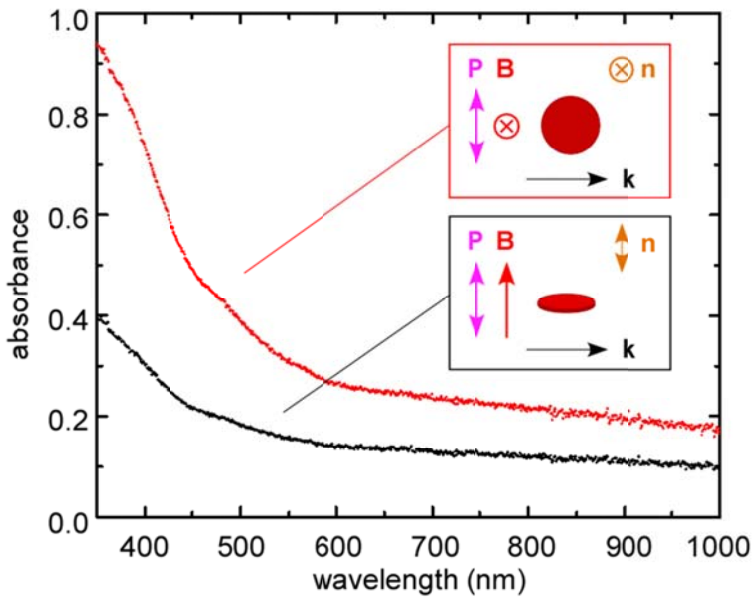

Supplementary Figure 2: Optical dichroism of disk-shaped BF nanoplates.

Optical absorbance of barium hexaferrite (ϕ = 0.003 in 50 μm thick cell) oriented by a 20 mT magnetic field applied either along or perpendicular to the probe beam **k** to induce alignment of the director **n**. The polarization direction **P** of the incident beam is fixed. The BF suspensions absorb strongly at short wavelength, making them appear red. The BF nanoplates are dichroic, absorbing more strongly for polarization in the plane of the nanoplates.

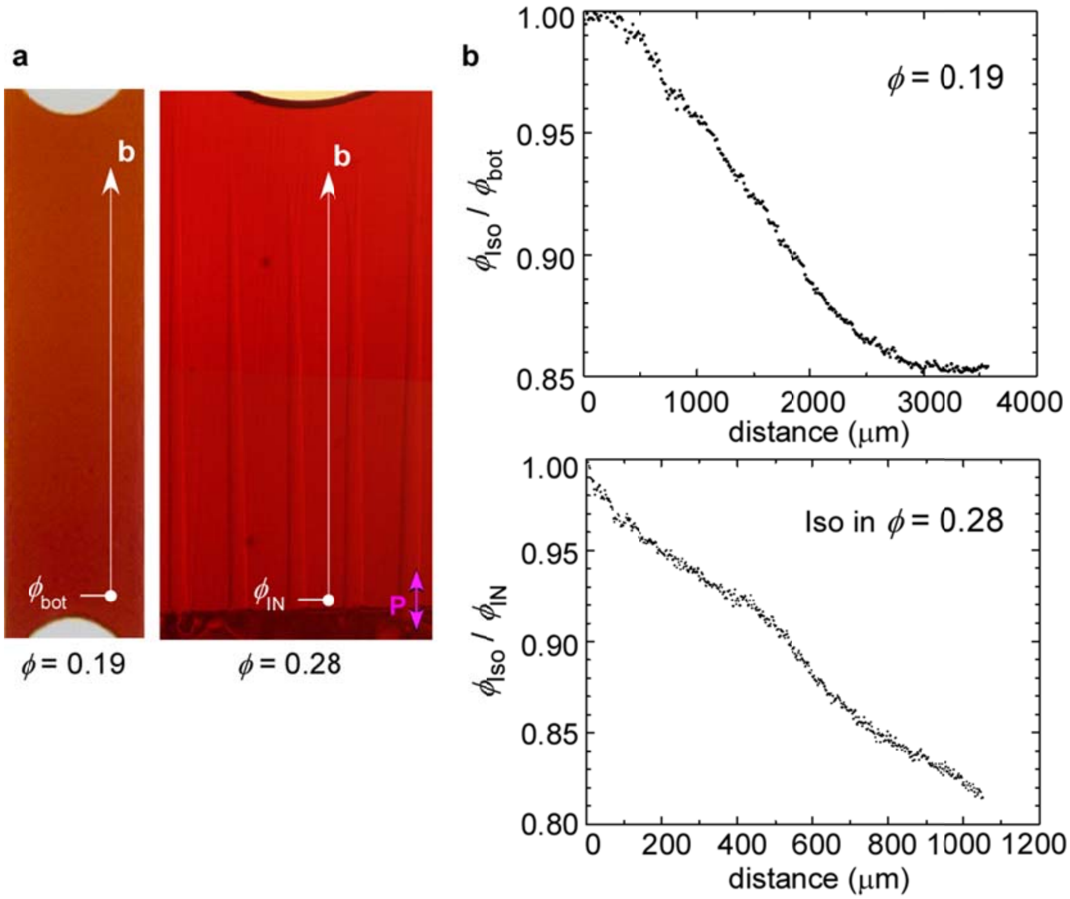

Supplementary Figure 3: Density gradient of BF nanoplates in the Iso phase.

(**a**) Typical textures obtained with a single optical polarizer (magenta, **P**) of the ϕ = 0.19 and ϕ = 0.28 suspensions equilibrated for two days in a vertical 0.05 mT magnetic field. (**b**) The density – expressed as the relative volume fraction $\phi_{Iso}(b)/\phi_{bot}$ for the ϕ = 0.19 suspension where ϕ_{bot} is the volume fraction at the bottom of the capillary, and $\phi_{Iso}(b)/\phi_{IN}$ for the ϕ = 0.28 suspension where ϕ_{IN} is the volume fraction just above the Iso/N_F interface – is plotted as a function of distance along the **b** axis. In both samples, the gravity-induced concentration gradient in the Iso phase is appreciable but small. Concentration differences of less than 20% were detected over their height of a few millimeters. The relative volume fraction is calculated from the intensity using $\phi_{Iso}(b)/\phi_{IN} = \ln(I_{Iso}(b)/I_o)/\ln(I_{IN}/I_o)$, where I_o, I_{IN}, and $I_{Iso}(b)$ are respectively the transmitted intensities of the BuOH solvent, of the Iso phase of the BF/BuOH suspension just above the Iso/N_F interface, and of the upper part of the Iso phase.

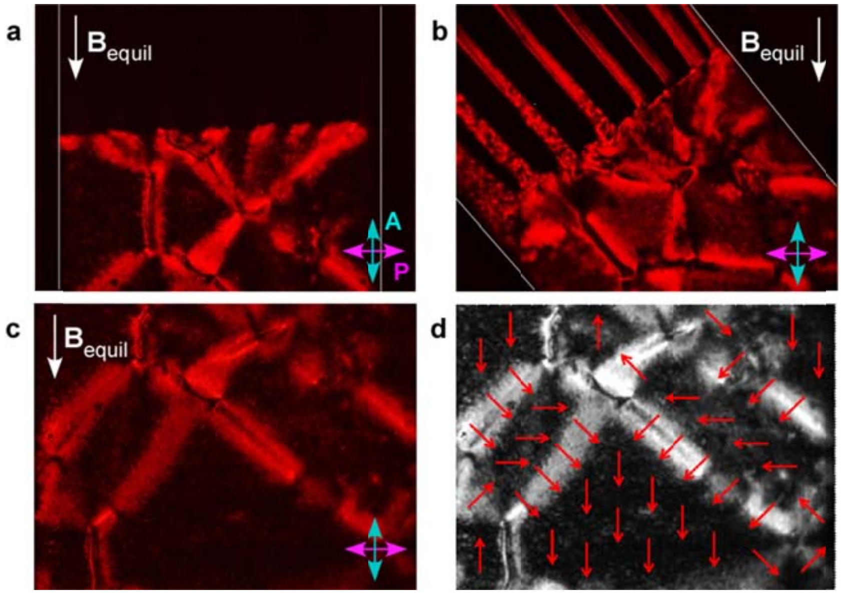

Supplementary Figure 4: Domain structure and interfacial spikes under 0.05 mT equilibrium magnetic field.

Capillary BF/BuOH cell at ϕ = 0.28 with an Iso/N_F interface equilibrated for several days in an external magnetic field B_{equil} ~ 0.05 mT, directed downward. (**a**) With the analyzer oriented parallel to $\mathbf{B}_{equil,}$ the upper part of the capillary appears dark. (**b**) Rotation of the cell reveals nematic spikes in the Iso phase, which have grown from the interface. The nematic director in the spikes is initially along $\mathbf{B}_{equil}$ but begins to reorient when the cell is rotated, producing the disordered/mottled texture in the leftmost spikes. (**c**) In accordance with Fig. 3b, the block domain with magnetization along the applied downward field has grown to be the largest. (**d**) Magnetization field of the domain structure in (**c**).

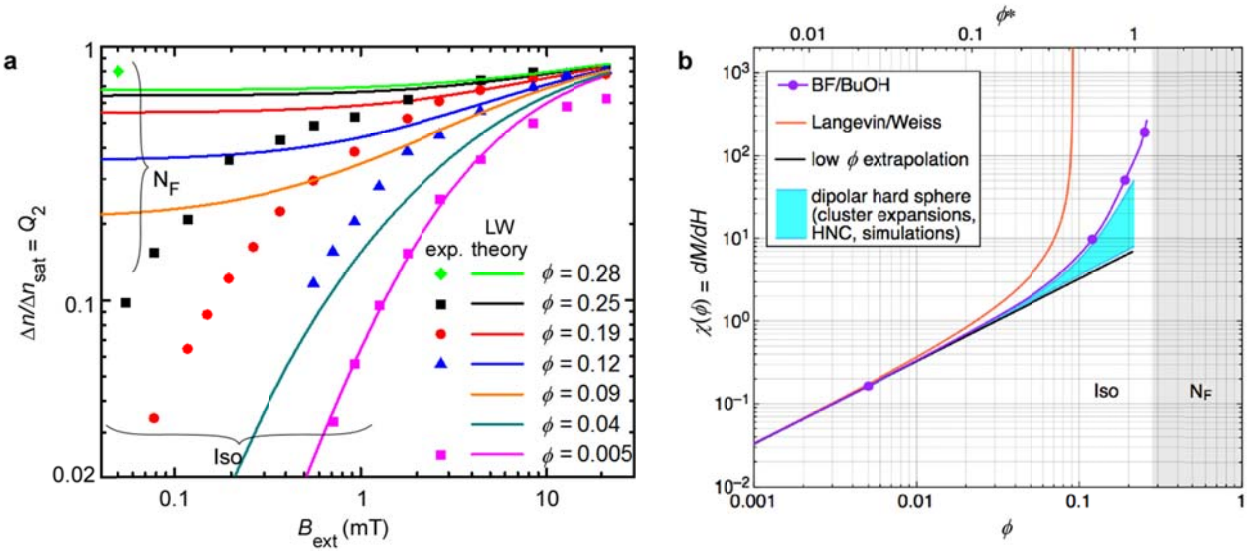

Supplementary Figure 5: Magnetic field induced birefringence and ordering of the Iso and N_F nanoplate suspensions.

(**a**) (*symbols*) Experimental birefringence $\Delta n/\Delta n_{sat}$ versus B_{ext} for four BF/BuOH suspensions in the Iso phase at concentrations ϕ = 0.25, 0.19, 0.12 and 0.005, and in the N_F phase at ϕ = 0.28 and small B_{ext}. (*solid lines*) Calculated $\Delta n/\Delta n_{sat}$ versus B_{ext} curves from the LW mean-field theory for polydisperse nanoplate suspensions with volume fractions shown in the legend (Supplementary Eq. 12). The mean magnetic moment $m_o = 2\times10^{-18}$ A·m^2 is obtained from the best fit to the ϕ = 0.005 suspension, giving a dipolar coupling constant $\lambda = \mu_o m_o^2/(4\pi D^3 k_B T) = 0.9$. (**b**) (*symbols*) Experimental values of initial (low field) susceptibility χ versus ϕ, derived from data in (**a**) using Supplementary Eqs. 11, 5, and 4 for the four Iso suspensions at concentrations ϕ = 0.25, 0.19, 0.12 and 0.005 under low fields. On the top axis indicated is $\phi^* = \nu\pi D^3/6 = 2D\phi/3t$, the effective volume fraction of spheres swept out by rotational diffusion of the discs. The plot shows calculated χ versus ϕ and ϕ^* for λ = 0.9 using several theoretical and simulation methods to account for orientational and positional correlations, as reviewed in Ref. 1. The models all have $\chi = 8\phi^*\lambda$ in the limit of low concentration to match the ϕ = 0.005 susceptibility data. The LW mean-field theory then predicts the Iso/N_F transition at a value of ϕ that is too small, as is also evident in (**a**). The monodisperse dipolar hard sphere models of Ref. 1 are distributed in the cyan region, with the largest χ coming from the Reference Limited Hypernetted Chain model of Patey[2], which is also the closest to the experimental data. This supports the notion that at lower concentrations the plates may be approximated as interacting dipolar hard spheres.

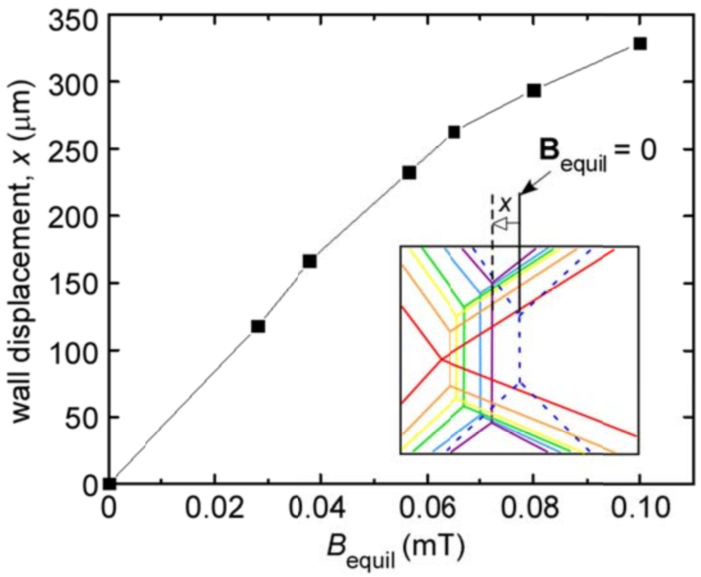

Supplementary Figure 6: Domain wall displacement under applied magnetic field.

Neighboring magnetic domains with opposite magnetization directions separated by a domain wall change size as the wall shifts under an applied magnetic field, $\mathbf{B}_{equil}$, applied parallel to the magnetization direction of the domain on the right-side of the wall (Fig. 3b). The linear dependence of the position of the wall on field strength indicates an elastic deformation of the wall system in response to field-induced stresses $\sigma_M \propto U_M = -\mathbf{M} \cdot \mathbf{B}_{equil}$. The linear response constitutes compelling evidence for an equilibrium magnetization density in the field-free state. Insert: Domain boundary positions as a function of applied field strength: dashed, $B_{equil} = 0$; purple, $B_{equil} = 0.028$ mT; blue, $B_{equil} = 0.038$ mT; green, $B_{equil} = 0.057$ mT; yellow, $B_{equil} = 0.065$ mT; orange, $B_{equil} = 0.080$ mT; red, $B_{equil} = 0.10$ mT.

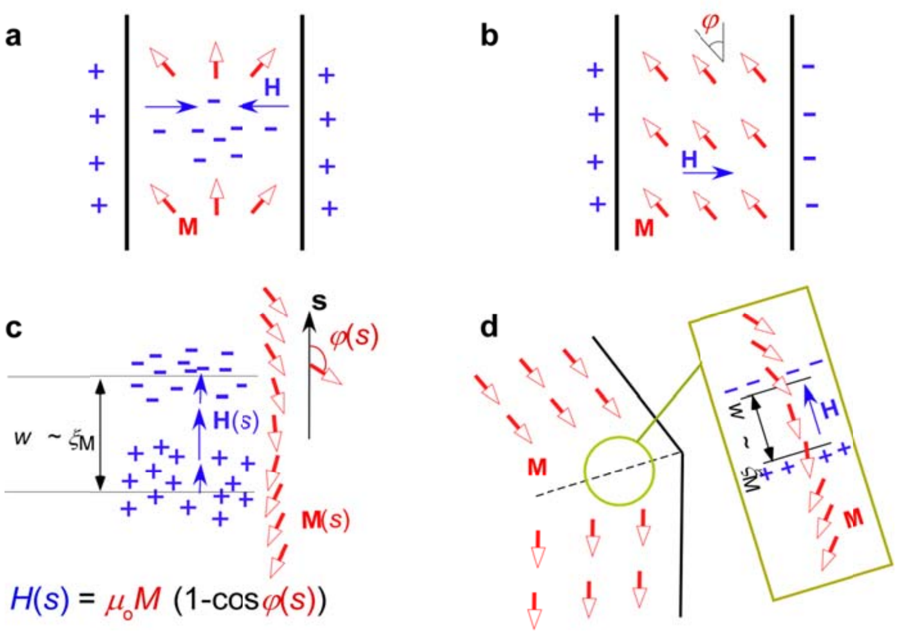

Supplementary Figure 7: Textures of M(r) at domain boundaries.

(**a**) Splay distortion of **M**(**r**) between infinite, parallel boundary sheets, showing the associated bulk magnetic charge $\rho_m(\mathbf{r}) = -\nabla\cdot\mathbf{M}(\mathbf{r})$ and surface magnetic charge $\rho_s(\mathbf{r}) = \mathbf{M}(\mathbf{r})\cdot\mathbf{s}(\mathbf{r})$, which generates an **H** field within the magnetized material. If the splay deformation is reduced to zero, then $H = 0$. (**b**) If **M**(**r**) is uniformly rotated away from being parallel to the infinite, confining boundary sheets by an angle φ, then the surface magnetic charge generates a uniform field $H = M\sin\varphi$ within the magnetized material. (**c**) Boundaries between two semi-infinite domains of uniform orientation. The boundary conditions cause the planar interface between the two domains to take the form of a two-dimensional splay/bend/splay wall of width $w \sim \xi_M = \sqrt{K/(\mu_0 M^2)}$ (K is the LC Frank elastic constant in the one-constant approximation), which is a magnetic counterpart of the case in ferroelectric LCs[3]. K may be taken to be comparable to the splay elastic constant because it is much larger than that for bend. This structure is a soliton-like solution[3] of Supplementary Eq. 20. **s** is the coordinate describing the displacement normal to the wall. (**d**) Structure of a ferromagnetic domain having a planar border with a discontinuous change of direction. The boundary condition prefers **M**(**r**) to be parallel to the border everywhere in order to reduce magnetic charge there. This generates semi-infinite domains of uniform **M**(**r**) that require a combination of splay and bend deformation to connect smoothly. The magnetic energy associated with splay-generated magnetic charge is minimized here by making the region of deformation as thin as possible. The splay/bend/splay structure of (**c**) is the local solution to this condition, creating a domain boundary that has the same local structure everywhere along a line that bisects the angle made by the kink in the border.

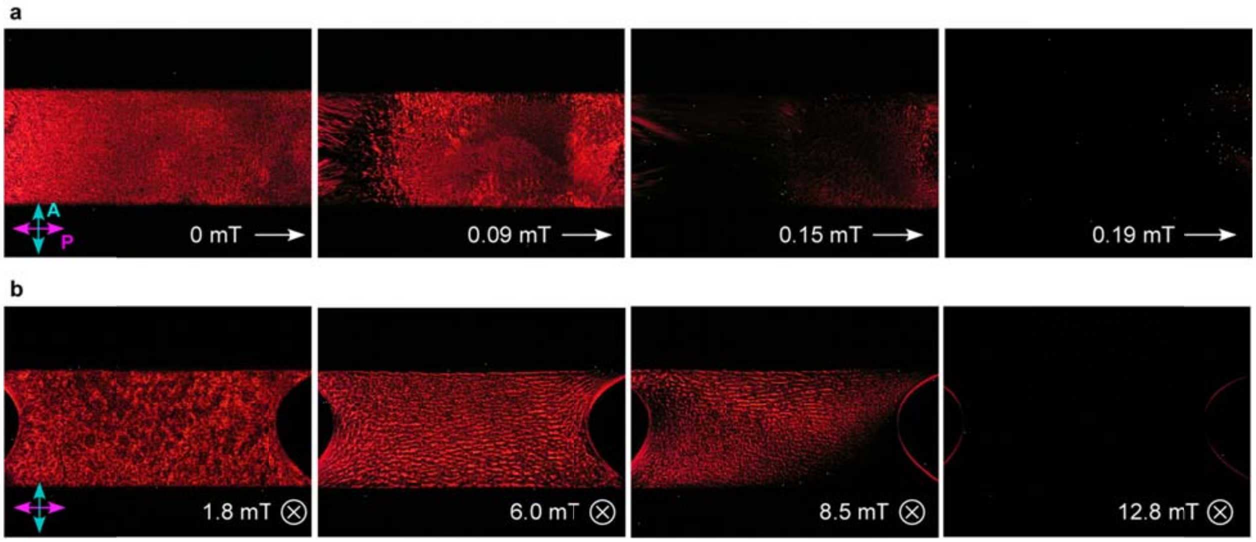

Supplementary Figure 8: Anisotropic alignment of BF/BuOH in external magnetic fields.

(**a**) $\mathbf{B}_{ext}$ parallel to the cell plane. An in-plane field $\mathbf{B}_{ext} \sim 0.2$ mT aligns a homogenized $\phi = 0.28$ sample to a bar-magnet-like monodomain. If this field is then reduced, the resulting magnetic charge on the poles of the bar magnet generate a demagnetizing field that reorients the LC into an array of small, randomly oriented domains, just as shown in the first image on the left. (**b**) $\mathbf{B}_{ext}$ normal to the cell plane. Substantial reorientation of **M** and **n** out of the cell plane requires a large field, $\mathbf{B}_{ext} \gtrsim 13$ mT.

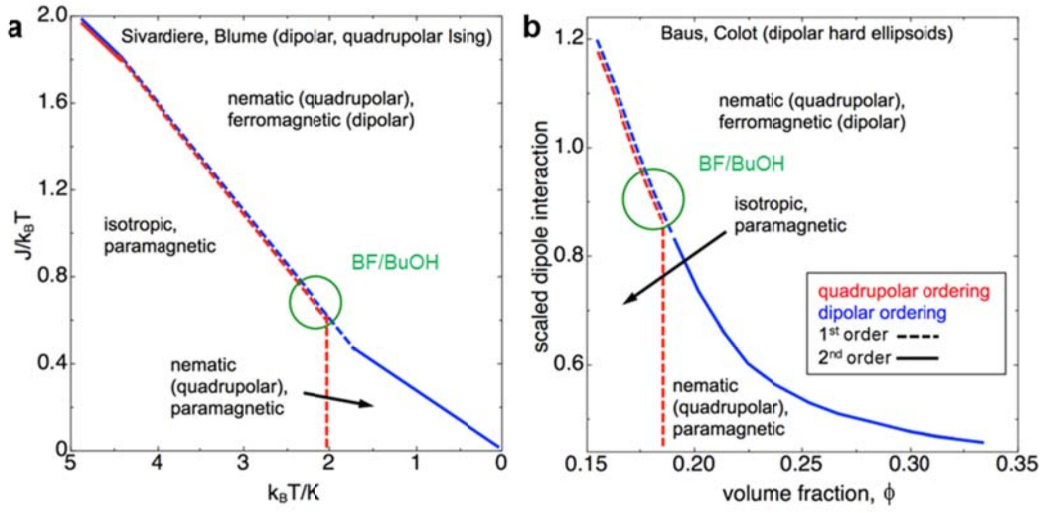

Supplementary Figure 9: Mean-field phase diagrams of model systems with dipolar and quadrupolar interactions.

(**a**) Sivardiere/Blume Ising model on a lattice with a Hamiltonian of nearest-neighbor dipolar and quadrupolar interactions[4]. J is the pair interaction energy of the dipolar term and K the interaction energy of the quadrupolar term. The phase diagram is calculated in the molecular field approximation. (**b**) Baus/Colot off-lattice model of hard ellipsoids of revolution with embedded dipoles[5]. ϕ is the volume fraction of the ellipsoids.

SUPPLEMENTARY NOTES

Supplementary Note 1: Field-induced magnetization and birefringence in the Iso phase

In order to assess the nature of the magnetic ordering in the Iso phase, $\Delta n(B_{ext})/\Delta n_{sat}$, where Δn_{sat} is the limiting Δn at high fields, is measured for several BF/BuOH concentrations, using 630 nm monochromatic light. The results are shown in Supplementary Fig. 5a. To analyze the data, we first tried to apply the standard Langevin-Weiss (LW) mean-field model[6], accounting for the polydispersity of the particle sizes, which enables calculation of Q_1 and Q_2 for a system of magnetic dipoles in thermal equilibrium. For a given diameter distribution function, the free parameters in the model are the temperature T (here always 298 K), the total particle number density ν, and the mean nanoplate magnetic moment m_o. For the purpose of this calculation, the nanoplates are divided into i = 23 species according to their diameters with a 5 nm bin size and low and high end cut-offs at 10 nm and 120 nm, respectively, following the fitted distribution function $f(D;\kappa,\tau) = D^{\kappa-1}e^{-D/\tau}/[\tau^{\kappa}\Gamma(\kappa)]$, where κ = 5.2 and τ = 9.2. In the absence of an applied magnetic field, the nanoplate moments in the Iso suspensions of orient randomly. When the Iso suspensions are subjected to an applied field, the magnetic torques interaction tend to align the magnetic moments of individual nanoplates with the field. If the field is strong enough, the magnetic moments of all the nanoplates will be completely aligned and the magnetization of the suspension will reach the saturation value M_{sat}

$$M_{sat} = \langle \nu m_o \rangle = \sum_i \nu_i m_i \, , \tag{1}$$

where the subscribed i represents the ith species.

When the magnetic field is not strong enough to completely align the magnetic moments of the nanoplates, thermal fluctuations tend to randomize the orientation of the nanoplates. The effective magnetic moment of a nanoplate is its component along the field direction, $m_i\cos\vartheta_i$, where ϑ_i is the angle between $\mathbf{m}_i$ and the applied magnetic field $\mathbf{B}_{ext}$. The field-induced magnetization of the suspension is the total effective magnetic moment per unit volume. It can be described by the first-rank order parameter $Q_{1i}(B_{ext})$

$$Q_{1i}(B_{ext}) = \langle \cos\vartheta_i \rangle = \frac{\int_0^{\pi/2} g(\vartheta_i)\cos\vartheta_i \sin\vartheta_i d\vartheta_i}{\int_0^{\pi/2} g(\vartheta_i)\sin\vartheta_i d\vartheta_i} \, , \tag{2}$$

with $g(\vartheta_i)$ being the orientational distribution function for the ith species. $g(\vartheta_i)$ can be expressed as

$$g(\vartheta_i) = \exp(m_i B\cos\vartheta_i / k_B T) , \tag{3}$$

where k_B is the Boltzmann constant and B *is* the total magnetic field. For convenience, we define

$$\gamma_i = m_i B / k_B T. \tag{4}$$

After integration, we obtain the so-called Langevin function

$$Q_{1i}(B) = \langle \cos\vartheta_i \rangle = \coth\gamma_i - \frac{1}{\gamma_i}. \tag{5}$$

The total magnetic field is a sum of the applied magnetic field B_{ext} and the internal magnetic field from the magnetized suspension

$$B = B_{ext} + \frac{1}{3}\mu_o \sum_i M_i , \tag{6}$$

where the second term describes the local magnetic field on a nanoplate in a spherical cavity of surrounding nanoplates. Taking all the species in a suspension into consideration, the overall first-rank order parameter is then

$$Q_1 = \frac{\langle M \rangle}{\langle v m_o \rangle} = \frac{\sum_i M_i}{\sum_i v_i m_i}. \tag{7}$$

The field-induced birefringence, $\Delta n(B_{ext})$, can be expressed in terms of the second-rank order parameter Q_2 as

$$\Delta n_i = \alpha\pi v_i D_i^2 Q_{2i} / (8 n_m), \tag{8}$$

where α is the optical polarizability anisotropy per unit area of the nanoplates, n_m is the mean refractive index of the solution, and

$$Q_{2i} = \left\langle \frac{3\cos^2\vartheta_i - 1}{2} \right\rangle. \tag{9}$$

The mean value of $\cos^2\vartheta_i$ is obtained by integrating over the entire distribution

$$\langle \cos^2\vartheta_i \rangle = \frac{\int_0^\pi g_i(\vartheta_i)\cos^2\vartheta_i \sin\vartheta_i d\vartheta_i}{\int_0^\pi g_i(\vartheta_i)\sin\vartheta_i d\vartheta_i}, \tag{10}$$

which yields

$$Q_{2i} = 1 - \frac{3}{\gamma_i}\coth\gamma_i + \frac{3}{\gamma_i^2}. \tag{11}$$

When the field is strong enough that all the nanoplates are perfectly aligned, $Q_{2i} = 1$ and $\Delta n = \Delta n_{\mathrm{sat}}$. Taking all the species in the suspension into consideration, the overall second-rank order parameter is

$$Q_2 = \frac{\Delta n}{\Delta n_{\mathrm{sat}}} = \frac{\sum_i v_i D_i^2 Q_{2i}}{\sum_i v_i D_i^2}. \quad (12)$$

For low concentration suspensions, the magnetic coupling between nanoplates should be weak and the LW theory a reasonable approximation. For a $\phi = 0.005$ suspension, we find that $m_{\mathrm{o}} = 2\times10^{-18}$ A·m^2. This is very close to the value of 3×10^{-18} A·m^2 measured[7] for aligned and dried colloidal nanoplates with a mean diameter of 70 nm.

Supplementary Note 2: Low-field susceptibility of the Iso phase

To further understand the magnetization process in the nanoplate suspension system, we measured the field-induced birefringence $\Delta n(B_{\mathrm{ext}})/\Delta n_{\mathrm{sat}}$ versus B_{ext} in Iso suspensions of different concentration ϕ, shown in Supplementary Fig. 5a. From these data we extracted the initial (low-field) magnetic susceptibility, $\chi = \mathrm{d}M/\mathrm{d}H$, using the response at low fields along with Supplementary Eqs. 11, 5 and 4, under the assumption of monodisperse particles. Supplementary Fig. 5b shows the resulting χ (symbols) and the prediction of the LW mean field model. Both Supplementary Figs. 5a and b indicate that if the LW model is adjusted to fit χ in the limit of low ϕ, then the predicted Iso/N$_{\mathrm{F}}$ transition occurs at a much lower concentration than observed experimentally.

Since here the nanoplates are in the Iso phase, we may consider them to be discs with orientational diffusion, sweeping out volumes obtained by spinning the average size disc about its diameter and in doing so behaving like hard spheres, as in an Onsager isotropic phase. Pursuing this notion, we indicate in Supplementary Fig. 5b the range of susceptibilities obtained from several theoretical models for the susceptibility of monodisperse dipolar hard spheres[1], which account for the dipolar coupling and correlation in different ways. The equivalent spherical volume fraction ϕ^* is related to the nanoplate volume fraction ϕ by $\phi^* = v\pi D^3/6 = 2D\phi/3t$. These models all behave at low ϕ as the same distribution of independent plates, giving the same $\chi(\phi) \propto \phi$, and are therefore all scaled in the same way to match the experiment at low ϕ by setting m_o to 2×10^{-18} A·m^2. The dipolar coupling constant[1] used for the dipolar hard spheres curves in Supplementary Fig. 5b is $\lambda = \mu_{\mathrm{o}} m_{\mathrm{o}}^2/(4\pi D^3 k_{\mathrm{B}} T) = 0.9$.

The range of susceptibilities obtained from the dipolar hard sphere models in Fig. 4 of Ref. 1 are indicated by the cyan-shaded region in Supplementary Fig. 5b. At the low limit of the cyan susceptibility range is the Onsager approximation for dipolar spheres[8], and at the high limit the Reference Limited Hypernetted Chain model by Patey[2], the latter providing a reasonable qualitative description of $\chi(\phi)$. The concentration variable ϕ^* is that typically used in models of liquid crystal ordering by the steric interaction of discs, being defined such that $\phi^* \sim 1$ at the concentration where the effective spherical volumes occupied by nanoplates start to overlap each other, which is where the Onsager LC ordering of discs occurs. Thus, as ϕ^* approaches 1, $\chi(\phi)$ increases faster than the dipolar hard sphere models, which we interpret to be due to enhanced correlations between the discs coming from their flat shape, beyond that arising from the magnetic interaction. Note that for monodisperse hard spheres, the value of ϕ^* can never exceed ~0.74, which is the upper limit of the packing fraction of spheres. However, for the case of our nanoplates, $\phi^* = 1$ corresponds to a nanoplate volume fraction of only $\phi \sim 0.22$, and above this concentration is where the Onsager excluded volume theory predicts that an Iso to nematic phase transition occurs[9]. Numerical simulations of polydisperse and charged disc suspensions[10-12] show that an Iso/Nematic transition starts to take place at $\nu\langle D^3\rangle = 3.2 \sim 4.0$. Considering that the volume of a nanoplate is $\pi\langle D^2 t\rangle/4$, and assuming the size distribution shown in Supplementary Fig. 1b, we can calculate the equivalent Iso/N_F phase transition concentration $\phi = 0.27 \sim 0.34$, which corresponds very well with our experimental observations. Thus, at $\phi \sim 0.28$ the BF/BuOH system is near an Onsager-type nematic ordering transition that enhances translational entropy by orienting hard discs to reduce their mutual excluded volume. This nematic ordering transition leads to the dramatic increase of χ and to the formation of the N_F phase.

Supplementary Note 3: Orientational order parameters of N_F BF/BuOH suspensions from X-ray diffraction

The birefringence and dichroism of the domains in the N_F phase indicate orientational ordering of the nanoplate planes. The order parameter of this uniaxial N_F phase can be calculated from these intensity distribution functions as follows. The orientational order of plates in the N_F phase is described by an orientational distribution function $f(\beta)$, in which β is the angle between the axis of the plates and the macroscopic symmetry axis of the N_F phase, similar to studies on thermotropic liquid crystals with molecules that possess the cylindrical symmetry. Following the method first established by Leadbetter and Norris[13], the scattered intensities from a uniaxially aligned liquid crystal depends on the orientational distribution as follows:

$$I(\theta) = \int_{\beta=\theta}^{\pi/2} f(\beta) \frac{\sin\beta \sec^2\theta}{\sqrt{\tan^2\beta - \tan^2\theta}} d\beta, \tag{13}$$

where $I(\theta)$ is the azimuthal profile, as illustrated in Fig. 2d (insert). To find $f(\beta)$ from the above function, we follow the method developed by Davidson *et al* [14]. First, $f(\beta)$ is expanded as a Fourier series as follows

$$f(\beta) = \sum_{n=0}^{\infty} f_{2n} \cos^{2n}\beta. \tag{14}$$

By inserting this in Supplementary Eq. 13, we obtain, after integration,

$$I(\theta) = \sum_{n=0}^{\infty} f_{2n} \frac{2^n n!}{(2n+1)!!} \cos^{2n}\theta. \tag{15}$$

The nematic order can be described by the second-rank order parameter

$$Q_2 = \left\langle \frac{3\cos^2\beta - 1}{2} \right\rangle, \tag{16}$$

where

$$\left\langle \cos^2\beta \right\rangle = \frac{\int_0^{\pi/2} f(\beta)\cos^2\beta \sin\beta d\beta}{\int_0^{\pi/2} f(\beta)\sin\beta d\beta}. \tag{17}$$

Applying Supplementary Eq. 14, we obtain

$$\left\langle \cos^2\beta \right\rangle = \frac{\sum_{n=0}^{\infty} \frac{f_{2n}}{2n+3}}{\sum_{n=0}^{\infty} \frac{f_{2n}}{2n+1}}, \tag{18}$$

from which we may obtain the order parameter. $Q_2 = 1$ represents a perfectly oriented state, while $Q_2 = 0$ describes an isotropic state. It is found that $Q_2 = 0.8$ at *Location* N_F and $Q_2 = 0.4$ at *Location Iso/*N_F. The form factor of the nanoplates, which broadens the distribution by the ratio $(2/D)/(2\pi/d) \sim 7^{\circ}$, was ignored in this estimate.

Supplementary Note 4: Ferromagnetic nematic magneto-elastic deformation energy

We analyze the textural features of the ferromagnetic nematic phase as phenomena resulting from the combined effects of magnetostatic and Frank nematic elastic energies, described by

$$U = \frac{\mu_0 M^2}{2} \int \mathrm{d}\mathbf{r}\mathrm{d}\mathbf{r}' \left[\nabla \cdot \mathbf{n}(\mathbf{r}) \nabla \cdot \mathbf{n}(\mathbf{r}') + (\mathbf{n}(\mathbf{r}) \cdot \mathbf{s}(\mathbf{r_s})\delta(\mathbf{r} - \mathbf{r_s}))(\mathbf{n}(\mathbf{r}') \cdot \mathbf{s}(\mathbf{r_s}')\delta(\mathbf{r}' - \mathbf{r_s}'))\right] |\mathbf{r} - \mathbf{r}'|^{-1} \tag{19}$$

$$+\int d\mathbf{r}\frac{1}{2}[K_S(\nabla\cdot\mathbf{n}(\mathbf{r}))^2+K_T(\mathbf{n}(\mathbf{r})\cdot\nabla\times\mathbf{n}(\mathbf{r}))^2+K_B(\mathbf{n}(\mathbf{r})\times\nabla\times\mathbf{n}(\mathbf{r}))^2]+\int d\mathbf{r}\mathbf{M}(\mathbf{r})\cdot\mathbf{B}(\mathbf{r})_{ext},$$

We assume here that the magnetic moments $\boldsymbol{m}_o$ are fixed to be normal to the colloidal plates, making $\mathbf{M}(\mathbf{r})$ and $\mathbf{n}(\mathbf{r})$ locally parallel everywhere, *i.e.*, $\mathbf{M}(\mathbf{r}) = M\mathbf{n}(\mathbf{r})$. The nematic Frank elastic energy includes the usually splay, bend, and twist deformation terms. The self-interaction of the magnetic dipole field has been written in terms of the bulk and surface magnetization charge densities $\rho_m(\mathbf{r}) = -\nabla\cdot\mathbf{M}(\mathbf{r}) = -M\nabla\cdot\mathbf{n}(\mathbf{r})$ and $\rho_s(\mathbf{r}_s) = M\mathbf{n}(\mathbf{r})\cdot\mathbf{s}(\mathbf{r}_s)\delta(\mathbf{r}-\mathbf{r}_s)$, respectively.

As discussed in the main text, strong magnetic charge effects confine $\mathbf{M}(\mathbf{r})$ in the sample of Fig. 3 to be parallel to the **a**,**b** plane, with the texture corresponding to a rotation of $\mathbf{M}(\mathbf{r})$ about **c**. In the case that the characteristic length $\xi_M = \sqrt{K/(\mu_o M^2)}$ is small compared to the sample thickness L, we can take the sample to be infinite in thickness and uniform along **c**, giving an orientation field φ that is 2D. For an in-plane sinusoidal reorientation of $\mathbf{M}(\mathbf{r})$ of wavevector **q**, Supplementary Eq. 19 then yields a magnetic space charge modulation giving an energy/volume

$$U_{\mathbf{q}} = \frac{1}{2}[\mu_o M^2(\cos\psi)^2 + K_B(q\sin\psi)^2 + K_S(q\cos\psi)^2]\ |\delta\varphi_{\mathbf{q}}|^2, \tag{20}$$

where ψ is the angle between **q** and $\delta\mathbf{n} = \mathbf{c}\times\mathbf{n}$, showing explicitly the dominance of magnetostatic splay interactions at long length scale (small q). This energy exhibits a wavevector dependence such that magnetostatic energy dominates Frank elasticity and suppresses splay deformation ($\mathbf{q}\perp\mathbf{M},\mathbf{n}$) on length scales longer than ξ_M. At shorter length scales, the LC can resist the magnetic torques. This behavior can be seen explicitly for the 3D sample with $\mathbf{M}(\mathbf{r})$ in the **a**,**b** plane and exhibiting a 2D rotation field φ about **c**, by using the applicable torque balance equation coming from Supplementary Eq. 19. For the splay/bend/splay wall shown in Fig. 3e and Supplementary Fig. 7c,d, spatial dependence of $\varphi(s)$ along a single coordinate **s** describing the displacement normal to the wall, we have

$$K\frac{\partial^2\varphi}{\partial s^2} = \mu_o\mathbf{M}\times\mathbf{H} = \mu_o M^2\sin\varphi(s)[\cos\varphi_o - \cos\varphi(s)]\,, \tag{21}$$

where we have taken $K_S = K_B = K$, **H** is generated by the magnetic charge, and φ is in the range $\varphi_o < \varphi < \varphi_o + \pi$. The soliton-like analytic solution for the wall structure is sketched in Supplementary Fig. 7c, a result initially obtained for the analogous electric case[3]. Assuming K the value of the splay constant $K_S = 6k_BT/D = 5\times10^{-13}$ N, obtained from Monte Carlo simulation of cut-spheres[15] with thickness-over-diameter ratio of 1/10 and $Q_2 \sim 0.8$, we find $\xi_M \sim 0.1$ μm. Nematic regions substantially larger than this, such as those shown in Fig. 3, are thus expected to have uniform orientation of $\mathbf{M}(\mathbf{r})$ and $\mathbf{n}(\mathbf{r})$ [16].

In the other limiting case, of a thin sample that is uniform along **c**, with spatial variation φ in the **a**,**b** plane, but where ξ_M is larger than with the sample thickness L, the normal mode energy/area becomes:

$$U_{\mathbf{q}} = \frac{1}{2}\left[\mu_o (ML)^2 |q\cos\psi| + K_B L(q\sin\psi)^2 + K_S L(q\cos\psi)^2\right] \, |\delta\varphi_{\mathbf{q}}|^2. \tag{22}$$

The crossover length in this case is $\xi_{ML} = K/(\mu_o M^2 L)$, analogous to the result obtained for freely suspended ferroelectric LC films[16-18].

Analysis based on Supplementary Eq. 19 shows that for a slab of infinite area, the magnetic energy of a uniform **M** field free to orient in any direction is lowest when **M** is parallel to the plane of the slab, in which case: (***i***) there is a uniform magnetic field $\mathbf{B}_M = \mu_o\mathbf{M}$ everywhere in the slob, and the macroscopic mean field in the magnetized material, oriented parallel to and stabilizing **M**, yields the lowest achievable magnetic energy ($U_M = -\mu_o M^2$). An estimate of $\mathbf{B}_M$, assuming $m_o = 2\times10^{-18}$A·m^2, is $B_M = \mu_o M \sim \mu_o Q_1 \Sigma v_i m_i \sim 3\times10^4 \mu_o$ A·m^{-1} ~ 40 mT; (***ii***) $B = 0$ outside of the slab; and (***iii***) the magnetic energy increases harmonically as $\delta U_M(\varphi) = \mu_o M^2 \varphi^2/2$ for rotation φ of **M** out of the cell plane. An external field $\mathbf{B}_{ext\perp}$ applied normal to the cell plane couples to the magneticzation with an energy density $U_M(\varphi) = -MB_{ext\perp}\varphi$ and leads to a field-induced rotation of M, $\varphi(B_{ext\perp}) = B_{ext\perp}/\mu_o M$, minimizing the free energy density with regard to φ.

Supplementary Note 5: Comparison of textural behavior in the BF/BuOH and BF/5CB systems

Comparison of the relative magnitudes of the energy terms in Supplementary Eq. 20 for the BF/BuOH and BF/5CB nematics[7] shows that the magnetic dipole interaction (M^2) term is larger by a factor of ~10^5 in BF/BuOH because of the larger density of magnetic nanoplates. At the same time, the Frank elastic constants in BF/BuOH are smaller ($K_B \sim 1\times10^{-13}$ N, $K_S \sim 5\times10^{-13}$ N), relative to those of BF/5CB (K_B, $K_S \sim 5\times10^{-12}$ N). These ratios combine to make the characteristic length $\xi_M = \sqrt{K/(\mu_o M^2)}$, expressing the balance between Frank elastic and magnetostatic torques, much smaller than the cell thickness. The result is an array of large local domains, separated by walls of dimension ξ_M that can respond independently to applied field. If the field is applied transverse to **M**, a uniform reorientation results. However, if, as is the case of Fig. 3c, the field is applied in the –**M** direction, depending on their initial local tilt, the domains

reorient in random directions, which is a distinctive feature of the regime where ξ_M is much smaller than the sample thickness.

In the BF/5CB case, the length ξ_M is much larger than the cell thickness, meaning that the M^2 self-interaction is irrelevant and the field response is determined by the balance of Frank elasticity and the interaction with $\boldsymbol{B}_{ext}$, yielding, for example, a Fréedericksz transition if $\boldsymbol{B}_{ext}$ is applied antiparallel to **M**, with a threshold where B_{ext} is large enough to reduce the magnetic coherence length $\xi_B = \sqrt{K/(MB_{ext})}$ to the cell thickness.

Supplementary Note 6: Analogy to high-polarization ferroelectric liquid crystals

The scenario of uniform blocks of magnetization separated by sharp domain walls is an example of "orientational fracture", similar to that found[17] in -1 topological defects in the director field structure and textures of thermotropic ferroelectric smectic C liquid crystals with large permanent polarization P. In this case, the polarization charge $\rho_p(\mathbf{r}) = -\nabla\cdot\mathbf{P}(\mathbf{r})$ produced by splay deformation of the polarization density $\mathbf{P}(\mathbf{r})$ field is sufficiently costly in energy that splay of $\mathbf{P}(\mathbf{r})$ is expelled from the bulk of a texture, rather being confined to narrow, **P**-stabilized 1/2 rotation walls of width $w \sim \xi_E = \sqrt{K_S/(\varepsilon_o P^2)}$ separating "block" domains of uniform $\mathbf{P}(\mathbf{r})$. Elastic energy functions of the form of Supplementary Eq. 19 have been employed extensively to describe such behavior in the absence of bulk free-charge screening of the polarization charge[19]. In the electric case, observation of the self-interaction effects of **P** require the polarization to be large enough to overcome the screening effects of free charge, from ions in solution, for example. Free charge on surfaces can also control the orientation of $\mathbf{P}(\mathbf{r})$ [18]. Since there are no magnetic free charges, the bulk and surface magnetic charges are only dipolar in nature, their self-interactions are always present, and there are always equal amounts of positive and negative magnetic charges.

SUPPLEMENTARY REFERENCES

1. Huke, B. & Lücke, M. Magnetic properties of colloidal suspensions of interacting magnetic particles. *Rep. Prog. Phys.* **67,** 1731-1768 (2004).

2. Patey, G. N. An integral equation theory for the dense dipolar hard–sphere fluid. *Mol. Phys.* **34,** 427-440 (1977).

3. Zhuang, Z., Maclennan, J. E. & Clark, N. A. Device applications of ferroelectric liquid-crystals - importance of polarization charge interactions. *Proc. SPIE* **1080,** 110-114 (1989).

4. Sivardie.J & Blume, M. Dipolar and quadrupolar ordering in S=3/2 Ising systems. *Phys. Rev. B* **5,** 1126-1134 (1972).

5. Baus, M. & Colot, J. L. Ferroelectric nematic liquid-crystal phases of dipolar hard ellipsoids. *Phys. Rev. A* **40,** 5444-5446 (1972).

6. Reitz, J. R., Milford, F. J. & Christy, R. W. *Foundations of Electromagnetic Theory*. (Addison-Wesley, Mass., 1979).

7. Mertelj, A., Lisjak, D., Drofenik, M. & Copic, M. Ferromagnetism in suspensions of magnetic platelets in liquid crystal. *Nature* **504,** 237-241 (2013).

8. Onsager, L. Electric moments of molecules in liquids. *J. Am. Chem. Soc.* **58,** 1486-1493 (1936).

9. Onsager, L. The effects of shape on the interaction of colloidal particles. *Ann. Ny. Acad. Sci.* **51,** 627-659 (1949).

10. Bates, M. A. & Frenkel, D. Nematic-isotropic transition in polydisperse systems of infinitely thin hard platelets. *J. Chem. Phys.* **110,** 6553-6559 (1999).

11. Bates, M. A. Influence of particle shape on the nematic—isotropic transition of colloidal platelet systems. *J. Chem. Phys.* **111,** 1732-1736 (1999).

12. Rowan, D. G. & Hansen, J. P. Salt-induced ordering in lamellar colloids. *Langmuir* **18,** 2063-2068 (2002).

13. Leadbetter, A. J. & Norris, E. K. Distribution functions in 3 liquid-crystals from X-ray-diffraction measurements. *Mol. Phys.* **38,** 669-686 (1979).

14. Davidson, P., Petermann, D. & Levelut, A. M. The measurement of the nematic order-parameter by X-ray-scattering reconsidered. *J. Phys. II* **5,** 113-131 (1995).

15. O'Brien, P. A., Allen, M. P., Cheung, D. L., Dennison, M. & Masters, A. Elastic constants of hard thick platelets by Monte Carlo simulation and virial expansion. *Soft Matter* **7,** 153-162 (2011).
16. Rosenblatt, C., Pindak, R., Clark, N. A. & Meyer, R. B. Freely suspended ferroelectric liquid-crystal films - absolute measurements of polarization, elastic-constants, and viscosities. *Phys. Rev. Lett.* **42,** 1220-1223 (1979).
17. Link, D. R., Chattham, N., Maclennan, J. E. & Clark, N. A. Effect of high spontaneous polarization on defect structures and orientational dynamics of tilted chiral smectic freely suspended films. *Phys. Rev. E* **71,** 021704 (2005).
18. Clark, N. A., Coleman, D. & Maclennan, J. E. Electrostatics and the electro-optic behaviour of chiral smectics C: 'block' polarization screening of applied voltage and 'V-shaped' switching. *Liq. Cryst.* **27,** 985-990 (2000).
19. Lu, M. H., Crandall, K. A. & Rosenblatt, C. Polarization-induced renormalization of the B_1 elastic-modulus in a ferroelectric liquid-crystal. *Phys. Rev. Lett.* **68,** 3575-3578 (1992).
